# Co-payments and adherence to endocrine therapy among Medicaid beneficiaries with breast cancer

**DOI:** 10.1007/s10549-026-08125-x

**Published:** 2026-11-04

**Authors:** Caitlin C. Murphy, Dyanna Gregory, Betsy Q. Cliff, Andrea C. Betts, Caroline L. Kirk, Sandi L. Pruitt, Anna Tavakkoli, Jasmin A. Tiro

**Affiliations:** 1 Section of Hematology and Oncology,Department of Pediatrics, The University of Chicago, 900 E 57th St., Rm.5118, Chicago, IL 60637 USA; 2 Department of Public Health Sciences, The University of Chicago, Chicago, IL USA; 3 UChicago Medicine Comprehensive Cancer Center, Chicago, IL USA; 4 Department of Internal Medicine, University of Texas Southwestern Medical Center, Dallas, TX USA; 5 Department of Health Promotion & Behavioral Sciences, UTHealth Houston School of Public Health, Dallas, TX USA; 6 Peter O’Donnell Jr. School of Public Health, University of Texas Southwestern Medical Center, Dallas, TX USA; 7 Harold C. Simmons Comprehensive Cancer Center, University of Texas Southwestern Medical Center, Dallas, TX USA

**Keywords:** Breast cancer, Endocrine therapy, Medicaid, Cost-sharing, Pharmacy benefit design

## Abstract

**Purpose:**

Prescription co-payments are associated with adherence to endocrine therapy among Medicare- and commercially insured-women, but the impact among Medicaid beneficiaries—who typically have no or minimal co-payments—is not well understood.

**Methods:**

We identified women (age ≥ 18 years) diagnosed with breast cancer between January 1, 2016 and December 31, 2018 who filled a prescription for endocrine therapy after diagnosis, with follow-up through December 31, 2019. Co-payment was based on the first prescription and standardized to a 30-day supply. We used quantity dispensed, days’ supply, and dispensing date to define adherence as a continuous measure of proportion of days covered and binary measure of +/- 80% days covered. We estimated associations with co-payment in linear and log binomial regression models limited to twenty states with observed within-state variation in co-payment.

**Results:**

10,307 Medicaid beneficiaries filled a prescription for endocrine therapy after breast cancer diagnosis. Mean (SD) co-payment amount was $1.85 ($1.28) and ranged from $0.17 to $8. Among 3,323 women living in states with variation in co-payment, there was no association between proportion of days covered and any co-payment (vs. none: β 0.70, *p* = 0.52) or co-payment amount (per dollar increase: β 0.03, *p* = 0.95), nor between adherence (≥ 80% days covered) and any co-payment (vs. none: PR 0.98, 95% CI 0.90, 1.08) or co-payment amount (per dollar increase: PR 0.99, 95% CI 0.96, 1.03).

**Conclusion:**

Most Medicaid-insured women with breast cancer had no or a nominal co-payment for endocrine therapy. Cost-sharing at these low levels was not associated with adherence.

## Introduction

Endocrine therapy, including tamoxifen and aromatase inhibitors (anastrozole, letrozole, exemestane), improves disease-free survival and reduces risk of breast cancer recurrence [1–5]. The American Society of Clinical Oncology [6] and National Comprehensive Cancer Network [7] recommend at least five—and up to 10—years of endocrine therapy for women diagnosed with early-stage, hormone receptor-positive breast cancer. Adherence to endocrine therapy is a well-documented challenge [8], and about half of women initiating therapy do not continue for the recommended duration. Numerous studies [9–13] have identified patient-level factors associated with adherence, including side effects, health beliefs, and knowledge, and interventions targeting these factors have had variable success [14, 15].

Policy-level factors, such as cost sharing, also influence medication adherence [16, 17] and may provide more effective and scalable levers for increasing access to and use of endocrine therapy. Growing evidence from Medicare-insured women with breast cancer suggests that partial or fully subsidized (< $10) compared to unsubsidized co-payments improve adherence to endocrine therapy [18, 19]. Similarly, the introduction of parity laws (i.e., laws limiting cost sharing for oral cancer medications in state-regulated insurance plans) [20] and generic aromatase inhibitors [21] has reduced out-of-pocket costs and subsequently improved adherence. Closing the Medicare Part D “doughnut hole [22]” has also increased access to oral cancer medications, including endocrine therapy [23, 24].

Low or subsidized co-payments may be particularly relevant for low-income women disproportionately affected by both breast cancer [25, 26] and the financial burden of cancer care [27, 28]. Medicaid is a joint federal-state program that provides health insurance coverage for low-income households and about 1 in 8 women (age 18–64 years) diagnosed with breast cancer in the United States [29]. Eligibility criteria and pharmacy benefits vary by state, particularly because some states expanded Medicaid eligibility under the Affordable Care Act. Prescription co-payments are statutorily capped at $8 [30]. Federal law [31] permits pharmacies to require co-payment as a condition for dispensing medication if the beneficiary: (i) has a family income > 100% of the federal poverty level (FPL); and (ii) is not part of an exempted group (e.g., pregnant women, children). For beneficiaries with a family income ≤ 100% FPL, medication may not be denied because of inability to pay; however, the co-payment remains a financial obligation and may influence subsequent decisions to fill or refill a prescription. Most states also maintain a preferred drug list and may additionally require prior authorization for medications not included on a preferred drug list and/or restrict the number of prescriptions per beneficiary.

To contextualize state-level differences in Medicaid pharmacy benefits, Table 1 summarizes expanded eligibility, preferred drug lists, and prescription co-payment requirements in each state [32, 33]. Thirteen states have no co-payment requirement, i.e., all prescriptions are provided for free, and the remaining 37 states plus Puerto Rico and the District of Columbia have co-payment requirements that vary by generic vs. brand, preferred vs. non-preferred, and cost. For example, North Dakota has no co-payment requirement for generic drugs and a $3 requirement for brand drugs, whereas North Carolina and Kansas require a $3 co-payment regardless of the generic vs. brand version. Although nominal, these co-payments may limit access to and deter use [34] of endocrine therapy.

**Table 1:** Co-payment requirements for Medicaid beneficiaries and preferred drug list by state

State	Co-Payment Requirements	Endocrine Therapy on Preferred Drug List	Medicaid Expansion
AK	$0.50 for drugs < $50, $3.50 for drugs ≥ $50	No	9/1/2015
AL	$0 - $3.90 based on drug cost	No	No
AR	Non-ACA Expansion: sliding cale ACA Expansion: none	No	1/1/2014
AZ	None	Yes	1/1/2014
CA	None	Yes	1/1/2014
CO	$3 with exceptions	No	1/1/2014
CT	None	Yes	1/1/2014
DC	$1 with exceptions	No	1/1/2014
DE	$0.50 - $3 based on drug cost, total not to exceed $15 per month	Yes	1/1/2014
FL	None	Yes	No
GA	$0.50 for preferred drugs, up to $3 for non-preferred drugs	Yes, except tamoxifen	No
HI	None	Yes	1/1/2014
IA	Non-ACA Expansion: $1 ACA Expansion: none	Yes	1/1/2014
ID	None	No	1/1/2020*
IL	$2 for generic drugs $3.90 for brand drugs	Yes	1/1/2014
IN	Non-ACA Expansion: $3 ACA Expansion: $4 for preferred drugs, $8 for non-preferred drugs	No	2/1/2015
KS	$3	No	No
KY	$1 for generic drugs and preferred brand drugs, $4 for other brand drugs	No	1/1/2014
LA	$0.50 for drugs ≤ $10, $1 for drugs ≤ $25, $2 for drugs ≤ $50, $3 for drugs > $50	Yes	7/1/2016**
MA	$3.65 with exceptions	Yes	1/1/2014
MD	$1 for generic and preferred brand drugs, $3 for other brand drugs	No	1/1/2014
ME	$3, total not to exceed $30 per month	Anastrozole and letrozole only	1/10/2019**
MI	Non-ACA Expansion: $1 for generic and preferred drugs, $3 for brand and non-preferred drugs ACA Expansion: $4 for generic and preferred drugs, $8 for brand and non-preferred drugs	No	4/1/2014
MN	$1 for generic drugs, $3 for brand drugs	No	1/1/2014
MO	$0.50 for drugs ≤ $10, $1 for drugs ≤ $25, $2 for drugs > $25	No	1/1/2021*
MS	$3 with exceptions	Yes, except tamoxifen	No
MT	$0 for generic drugs and select therapeutic classes, $4 for preferred brand drugs, $8 for non-preferred brand drugs	No	1/1/2016**
NC	$3	No	12/1/2023*
ND	$0 for generic drugs, $3 for brand drugs	No	1/1/2014
NE	$2 for generic drugs, $3 for brand drugs	Yes	10/1/2020*
NH	$1	No	8/15/2014
NJ	None	Yes	1/1/2014
NM	None	Yes, except exemestane	1/1/2014
NV	None	No	1/1/2014
NY	$1 for preferred brand, generic, and brand costing less than generic drugs, $3 for non-preferred brand drugs	Tamoxifen and letrozole only	1/1/2014
OH	$2 for selected brand drugs, $3 for drugs that require prior authorization	No	1/1/2014
OK	$4	Yes	7/1/2021*
OR	None	No	1/1/2014
PA	$0 for many drug classes, $1 for generic drugs, $3 for brand drugs	Yes	1/1/2015
PR	$1 for generic drugs, $3 for brand drugs	Tamoxifen and anastrozole only	55% FMAP
RI	None	No	1/1/2014
SC	$3.40	No	No
SD	$1 for generic drugs, $3.30 for brand drugs	No	7/1/2023*
TN	$1.50 for generic and preferred brand drugs, $3 for brand drugs	Yes	No
TX	None	Yes	No
UT	$4, not to exceed $20 per month	Yes	1/1/2020*
VA	$1 for generic drugs, $3 for brand drugs	No	1/1/2019**
VT	$1 - $3 based on Medicaid reimbursement rate	No	1/1/2014
WA	None	Yes	1/1/2014
WI	$1 for generic drugs, $3 for brand drugs, not to exceed $12 per provider per month	No	No
WV	$0 - $3 based on drug cost	No	1/1/2014
WY	$0.65 for generic drugs, $3.65 for brand drugs	No	No

NOTE: Co-payment requirements derived from KFF as of July 1, 2019 [49]

*Expanded Medicaid after study period, i.e., after December 31, 2019

**Expanded Medicaid during study period, i.e., between January 1, 2016 and December 31, 2019

We examined the association between co-payment amount and adherence to endocrine therapy in a large, racially and ethnically diverse sample of Medicaid-insured women diagnosed with breast cancer. By focusing specifically on Medicaid beneficiaries, we complement studies of Medicare- and commercially insured women with breast cancer [18, 19, 35–38] and generate policy-relevant evidence needed to provide affordable cancer care for low-income populations.

## Materials and methods

### Study population

We identified women (age ≥ 18 years) diagnosed with breast cancer between January 1, 2016 and December 31, 2018 using the Transformed Medicaid Statistical Information System (T-MSIS) Analytic Files (TAF). These data standardize enrollment and claims files across all 50 U.S. states, the District of Columbia, and Puerto Rico, including both fee-for-service and managed care plans. We used two criteria to identify women diagnosed with breast cancer, as described by Halpern and Schrag [39]. First, we required at least two claims with International Classification of Disease, Ninth (ICD-9: 174.X, 233.0, 238.3, 239.3) or Tenth (ICD-10: C50.X, D05.X, D48.X, D49.3) Revision diagnosis codes for breast cancer, on two separate dates and at least 30 days apart (*n* = 104,561). Second, we required a subsequent claim with a procedure code (either Current Procedural Terminology, ICD-9, ICD-10, or Diagnosis-Related Group codes, see Appendix) for mastectomy or breast conserving surgery (*n* = 28,779). Women with less than three months of continuous enrollment (*n* = 10,728) prior to diagnosis or age < 18 or ≥ 65 years (*n* = 751) were excluded because we could not establish a reliable claims history. We then identified women prescribed endocrine therapy as those with a prescription filled (≥ 14-day supply) after surgery (*n* = 12,498). We excluded women in North Carolina, South Carolina, Kentucky, Idaho, and Missouri due to incomplete claims or missing data on co-payment (*n* = 2,191). Supplementary Figure 1 illustrates selection into the sample (*n* = 10,307).

### Adherence to endocrine therapy

As recommended by the Pharmacy Quality Alliance [40] and National Association of Specialty Pharmacies [41], we measured adherence as the proportion of days covered: [(days covered by endocrine therapy / days eligible for endocrine therapy) x 100]. We summed days covered by endocrine therapy as the number of days supplied by a prescription. For example, a 30-day prescription filled on January 1st provided 30 days covered from January 1st through January 30th. We accounted for switching between doses or formulations by summing days covered by *any* endocrine therapy (i.e., tamoxifen, anastrozole, exemestane, or letrozole) rather than requiring fills of the same prescription. We then summed days eligible for endocrine therapy as the number of days from the first prescription fill through disenrollment from Medicaid, death, or December 31, 2019. We limited the study period through December 31, 2019 because many states enacted temporary changes in Medicaid pharmacy benefits (e.g., waived co-payment, increased prescription quantity limits) in response to the COVID-19 pandemic [42].

### Statistical analysis

We described characteristics of the sample, including age at diagnosis, race and ethnicity, year of diagnosis, family income relative to FPL, comorbidity, type of surgery (mastectomy vs. breast conserving surgery), year of endocrine therapy initiation, and type of endocrine therapy (selective estrogen receptor modulator [SERM] vs. aromatase inhibitor). We combined race and ethnicity to include Hispanic (of any race), non-Hispanic Asian American or Pacific Islander, non-Hispanic Black, non-Hispanic Other, non-Hispanic White, and Missing. Non-Hispanic Other included American Indian and Alaska Native, Multi-racial, and non-Hispanic (categorized by Medicaid). We reported Missing as a separate category because of state-level differences in the quality of race and ethnicity data in TAF [43]. For the same reason, we included Missing as a separate category for family income. We measured comorbidity within 3 months of diagnosis using a modified-version of the Elixhauser Comorbidity Index [44], a composite score based on non-cancer conditions identified from ICD-9 and ICD-10 diagnosis codes [45] and ranging from 0 (lower comorbidity) to 27 (higher comorbidity).

We estimated mean co-payment by age at diagnosis, race and ethnicity, family income, and type of endocrine therapy. Co-payment was derived from individual-level data in TAF and based on a woman’s first prescription fill for endocrine therapy, standardized to a 30-day supply.

To estimate the association between co-payment and adherence to endocrine therapy, we used four models that varied binary and continuous measures of both co-payment and adherence. In log binomial regression models, adherence was modeled as a binary measure of ≥ 80% of days covered [8] with (1) co-payment as a binary measure of any vs. none; and (2) co-payment as a continuous measure of dollar amount. In linear regression models, adherence was modeled as a continuous measure of proportion of days covered with binary and continuous measures of co-payment.

We selected confounders as those associated with adherence and/or co-payment [8]: age at diagnosis, race and ethnicity, comorbidity, year of endocrine therapy initiation, type of endocrine therapy, state (i.e., state-level policies governing the administration of Medicaid pharmacy benefits, including co-payment), and family income. Because some states do not require co-payment (Table 1), we limited the primary analysis to 20 states with observed within-state variation in any vs. no co-payment and included state as a fixed effect in each model to account for time-invariant differences in Medicaid policy and administration across states. Supplementary Table 1 summarizes characteristics of women in these states (*n* = 3,323). In a secondary analysis, we estimated the association between individual- and state-level co-payment (required vs. not required) and adherence (≥ 80% of days covered) in the total sample.

The Institutional Review Boards at the University of Texas Health Science Center at Houston and the University of Texas Southwestern Medical Center approved this study. All analyses were conducted in SAS version 9.4 (SAS Institute, Cary, NC). Statistical tests were two-sided; *p* < 0.05 indicated statistical significance.

## Results

We identified 10,307 Medicaid beneficiaries diagnosed with breast cancer and who filled a prescription for endocrine therapy (Table 2). Most women (57.7%) were prescribed an aromatase inhibitor (70.1% anastrozole, 5.2% exemestane, 24.8% letrozole). The sample was racially and ethnically diverse: 19.7% Hispanic, 16.3% non-Hispanic Black, and 8.0% non-Hispanic Asian American or Pacific Islander. Mean (SD) comorbidity score was 1.1 (1.6). Mean (SD) follow-up time was 2.5 (1.0) years.

**Table 2:** Characteristics of 10,307 Medicaid beneficiaries diagnosed with breast cancer and initiated endocrine therapy, 2016–2018

	*n*	(%)
Age at diagnosis – mean (SD)	51.3 (8.7)	
18–39 years	1,139	(11.1)
40–49 years	3,065	(29.7)
50–59 years	4,266	(41.4)
60–64 years	1,837	(17.8)
Race and ethnicity		
Hispanic (any race)	2,027	(19.7)
Non-Hispanic Asian	823	(8.0)
Non-Hispanic Black	1,682	(16.3)
Non-Hispanic Other^1^	105	(1.0)
Non-Hispanic White	4,106	(39.8)
Missing	1,564	(15.1)
Family income relative to federal poverty level		
0-100%	3,469	(33.7)
≥ 101	602	(5.8)
Missing	6,236	(60.5)
Comorbidity score – mean (SD)^2^	1.1 (1.6)	
0	4,994	(48.5)
1–3	4,472	(43.4)
≥ 4	841	(8.2)
Type of surgery		
Mastectomy	4,948	(48.0)
Breast conserving surgery	5,359	(52.0)
Time from diagnosis to initiation – mean (SD)	218.8 (188.9)	
< 30 days	787	(7.6)
30–90 days	1,744	(16.9)
91–180 days	2,623	(45.5)
> 180 days	5,153	(50.0)
Type of endocrine therapy initiated		
SERM	4,360	(42.3)
Aromatase inhibitor	5,947	(57.7)
Proportion of days covered – mean (SD)	76.4 (19.8)	
≥ 80%	5,681	(55.1)
Follow-up time – mean (SD)	2.5 (1.0)	

Abbreviations: SD, standard deviation; SERM, selective estrogen receptor modulator

^1^Non-Hispanic Other includes American Indian and Alaska Native, Multi-racial, and non-Hispanic, as reported by Medicaid

^2^Comorbidity score measured using the Elixhauser Comorbidity Index [44] within 3 months of diagnosis

Most women (90.1%, *n* = 9,289) had no co-payment. Among those with a co-payment (9.9%, *n* = 1,018), the mean (SD) amount was $1.85 ($1.28) and ranged from $0.17 to $8. Table 3 summarizes mean co-payment amount by age at diagnosis, race and ethnicity, family income, and type of endocrine therapy.

**Table 3:** Any co-payment and mean co-payment amount among 10,307 Medicaid beneficiaries diagnosed with breast cancer and initiated endocrine therapy, 2016–2018

	Any Co-payment		Co-payment Amount	
	*n*	(%)	Mean^1^	(SD)
Overall	1,018	(9.9)	1.8	(1.3)
Age at diagnosis – mean (SD)				
18–39 years	123	(10.8)	2.2	(1.3)
40–49 years	305	(10.0)	2.0	(1.2)
50–59 years	426	(10.0)	1.8	(1.2)
60–64 years	164	(8.9)	1.7	(1.4)
Race and ethnicity				
Hispanic (any race)	81	(4.0)	1.5	(1.1)
Non-Hispanic Asian	16	(1.9)	1.6	(1.3)
Non-Hispanic Black	144	(8.6)	1.4	(1.2)
Non-Hispanic Other^2^	3	(2.8)	0.2	(0.1)
Non-Hispanic White	336	(8.2)	1.5	(1.2)
Missing	438	(28.2)	2.4	(1.2)
Family income relative to federal poverty level				
0-100%	622	(17.9)	2.1	(1.4)
≥ 101	73	(12.1)	1.7	(1.4)
Missing	323	(5.2)	1.4	(0.9)
Type of endocrine therapy initiated				
SERM	441	(10.1)	1.9	(1.2)
Aromatase inhibitor	577	(9.7)	1.8	(1.3)

Abbreviations: SD, standard deviation; SERM, selective estrogen receptor modulator

^1^Mean (SD) co-payment estimated among women with any co-payment (*n* = 1,018, 9.9% of total)

^2^Non-Hispanic Other includes American Indian and Alaska Native, Multi-racial, and non-Hispanic, as reported by Medicaid

Mean (SD) proportion of days covered was 76.4% (19.8), and 55.1% of women (*n* = 5,681) were adherent (≥ 80% of days covered) to endocrine therapy. Adherence was similar by any vs. no co-payment. For example, mean (SD) proportion of days covered was 73.8% (22.0) among women with a co-payment compared to 76.7% (19.6) among women without a co-payment. Half (*n* = 509, 50.0%) of women with a co-payment were adherent (≥ 80% of days covered) compared to 55.7% of women without a co-payment.

Among 3,323 women living in states with observed variation in any vs. no co-payment, there was no association between adherence (≥ 80% of days covered) and any co-payment (vs. none: PR 0.98, 95% CI 0.90, 1.08) or co-payment amount (per dollar increase: PR 0.99, 95% CI 0.96, 1.03) (Table 4). Similarly, there was no association between proportion of days covered and any co-payment (vs. none: β 0.70, *p* = 0.52) or co-payment amount (per dollar increase: β 0.03, *p* = 0.95) (Table 5).

**Table 4:** Adjusted association between co-payment and adherence (≥ 80% of days covered) to endocrine therapy among 3,323 Medicaid beneficiaries diagnosed with breast cancer

Co-payment (binary)	Adjusted PR^1^	95% CI	*p*-value
None	Ref.		
Any	0.98	0.90, 1.08	0.71
Co-payment amount (continuous)	0.99	0.96, 1.03	0.74

Abbreviations: PR, prevalence ratio; CI, confidence interval; Ref, reference

^1^Adjusted for age at diagnosis, race and ethnicity, year of endocrine therapy initiation, comorbidity score, type of endocrine therapy, and family income

NOTE: Model limited to states with observed within-state variation in any vs. no co-payment: Alaska, Arizona, Colorado, Washington D.C., Georgia, Indiana, Kansas, Massachusetts, Maryland, Maine, Montana, New York, Oklahoma, Oregon, Puerto Rico, South Dakota, Tennessee, Vermont, Wisconsin, and West Virginia; state included as a fixed effect to account for time-invariant differences in policy and administration across states

**Table 5:** Adjusted association between co-payment and proportion of days covered by endocrine therapy among 3,323 Medicaid beneficiaries diagnosed with breast cancer

Co-payment (binary)	Adjusted ꞵ^1^	SE	*p*-value
None	Ref.		
Any	0.70	1.08	0.52
Co-payment amount (continuous)	0.03	0.46	0.95

Abbreviations: SE, standard error; Ref, reference

^1^Adjusted for age at diagnosis, race and ethnicity, year of endocrine therapy initiation, comorbidity score, type of endocrine therapy, and family income

NOTE: Model limited to states with observed variation in any vs. no co-payment: Alaska, Arizona, Colorado, Washington D.C., Georgia, Indiana, Kansas, Massachusetts, Maryland, Maine, Montana, New York, Oklahoma, Oregon, Puerto Rico, South Dakota, Tennessee, Vermont, Wisconsin, and West Virginia; state included as a fixed effect to account for time-invariant differences in policy and administration across states

In the total sample (*n* = 10,307), state-level co-payment was associated with adherence, i.e., living in a state with vs. without a co-payment requirement was associated with lower likelihood of adherence (PR 0.93, 95% CI 0.90, 0.97). There was no association with individual-level co-payment (any vs. none: PR 0.98, 95% CI 0.87, 1.08) or co-payment amount (per dollar increase: PR 1.00, 95% CI 0.96, 1.04) (Supplementary Table 2).

## Discussion

In a large, diverse sample of Medicaid beneficiaries diagnosed with breast cancer, less than two-thirds were adherent to endocrine therapy. Adherence was lower than adherence reported in prior studies of Medicaid-insured women (59% – 77%) [46, 47] but still consistent with the broader literature demonstrating suboptimal adherence across diverse populations and care settings [8]. Most women in our study had no or a nominal co-payment for endocrine therapy, and we observed no association between co-payment and adherence.

Our findings add to prior studies that have examined associations between co-payment and adherence to endocrine therapy among Medicare- and commercially insured women [18, 19, 35–38, 48]. The co-payment amounts examined in these studies range from ≥ $2 to ≥ $90, and in general, higher co-payments are associated with lower adherence. For example, co-payments ≥ $15 increased odds of non-adherence to aromatase inhibitors by up to five times among 15,000 Medicare-insured women with breast cancer [18]. Co-payments ≥ $10 for a 30-day supply and ≥ $30 for a 90-day supply have been associated with non-adherence among commercially insured-women [36, 37]. A more recent study of women insured by commercial or Medicare Advantage plans demonstrated that co-payments similarly increased risk of non-adherence to endocrine therapy across levels of household income [38]. Others have suggested that women with subsidized co-payments or who receive low-income subsidies (< $10) have improved adherence, in some cases surpassing adherence among higher-income women [19]. Like low-income subsidies, our study suggests co-payments in Medicaid may, on average, fall below a threshold that meaningfully affects adherence to endocrine therapy.

State-level policies governing the administration of Medicaid pharmacy benefits (e.g., preferred drug list, prior authorization, mail order dispensing, mandatory 90-day supply) may play a bigger role in adherence than co-payment. All U.S. states provide a pharmacy benefit as part of Medicaid, but they administer these benefits differently within federal guidelines about pricing and dispensing [49]. For example, Massachusetts [50] and Utah [51] require endocrine therapy to be dispensed as a 90-day supply after an initial 30-day supply, and New Mexico designates endocrine therapy as eligible for mail order [52]. Several studies demonstrate that adherence to medications for chronic conditions, including diabetes, hypertension, and hyperlipidemia [53, 54], increases with longer day supply and with the use of mail-order versus retail pharmacy [55–58]. Medicaid beneficiaries can also receive waivers or other forms of assistance [59], meaning that co-payment amounts recorded in claims may not reflect actual out-of-pocket costs. For example, Michigan reduces co-payments for beneficiaries who complete a health risk assessment or attest to a healthy behavior [60]. These state-level policies and practices highlight multiple levers within pharmacy benefit design that can be optimized to improve access to endocrine therapy while managing costs.

Patient-level factors, particularly treatment-related side effects [61–63], remain important determinants of adherence to endocrine therapy, and although not the focus of our study, are often cited as reasons for discontinuation or inconsistent use [64]. The frequency and severity of hot flashes, sexual dysfunction, weight gain, fatigue, and other side effects can affect the balance of treatment benefits and harms, even when endocrine therapy is readily accessible. Interventions to manage side effects, including cognitive behavioral therapy, selective serotonin reuptake inhibitors, and acupuncture [65], may therefore improve long-term adherence. These interventions may be especially critical for younger, premenopausal women with breast cancer – disproportionately represented among Medicaid beneficiaries – who experience side effects more acutely [66]. Other patient-level interventions have targeted medication-taking behaviors (e.g., text reminders, smart pill caps) but have demonstrated variable effectiveness [14].

Our study has some limitations. We identified breast cancer diagnoses with an algorithm [39] applied to administrative data and not from a cancer registry considered the gold standard. We also lacked information on hormone receptor status (i.e., eligibility for endocrine therapy) and required a prescription fill for endocrine therapy to define the analytic sample; therefore, we could not determine whether initiation differed by co-payment. Medication adherence measured in administrative data may overestimate true medication-taking behavior, i.e., administrative data cannot discern if a medication is taken as prescribed, although prior studies report high concordance between claims-based measures and pill counts [67]. Likewise, the reasons for non-adherence are not captured in administrative data, and some women may decide to pause or discontinue endocrine therapy in collaboration with their care team (e.g., to pursue pregnancy) [68]. The study period was limited to four years, with a mean follow-up of about two years, precluding assessment of adherence over the recommended 10-year course of endocrine therapy [5]. This relatively short follow-up is expected given the frequency of Medicaid disenrollment and coverage disruptions among patients with cancer [69] and underscores the challenges of evaluating long-term health outcomes in this population. We did not capture prescriptions for other medications, and co-payments for these prescriptions may indirectly affect adherence to endocrine therapy, particularly in states that limit monthly co-payments or the number of prescriptions per beneficiary [70]. Finally, there may be unobserved differences between women with and without a co-payment, and we cannot rule out residual confounding or establish causality.

These limitations may be outweighed by the important strength of our study: a large, racially and ethnically diverse sample of Medicaid-insured women with breast cancer. To date, studies examining the association between co-payment and adherence to endocrine therapy have been conducted among Medicare- or commercially insured women [18, 19, 35–38, 48], and we provide complementary evidence in an understudied, low-income population.

## Conclusion

In summary, we observed no association between co-payments and adherence to endocrine therapy among Medicaid-insured women with breast cancer living in states with within-state variation in co-payment. These findings suggest that cost-sharing required in many Medicaid programs may not be a barrier to adherence. Future efforts to improve adherence in this population may therefore benefit from focusing on other aspects of pharmacy benefit design, such as dispensing practices or fill synchronization [38], the impact of Medicaid “unwinding” and new work requirements [71], as well as managing treatment-related side effects. Identifying strategies that address both policy and patient-level determinants of adherence will be important for ensuring that low-income women receive the long-term benefits of endocrine therapy.

## Data Availability

The data that support the findings of this study are available from the Centers for Medicare & Medicaid Services (CMS) through the Research Data Assistance Center (ResDAC). Restrictions apply to the availability of these data, which were used under a data use agreement for this study and are not publicly available. Data may be obtained directly from CMS through ResDAC by qualified researchers who meet the requirements for access to these data.

## Abbreviations

CI: Confidence interval
ICD: International Classification of Disease
PR: Prevalence ratio
SD: Standard deviation
